# Association between *Tumor necrosis factor*-*alpha* gene polymorphisms and prostate cancer risk: a meta-analysis

**DOI:** 10.1186/1746-1596-9-74

**Published:** 2014-03-25

**Authors:** Liping Ma, Jiangyang Zhao, Taijie Li, Yu He, Jian Wang, Li Xie, Xue Qin, Shan Li

**Affiliations:** 1 Department of Clinical Laboratory, First Affiliated Hospital of Guangxi Medical University, Nanning, Guangxi Zhuang Autonomous Region 530021, China

**Keywords:** Tumor necrosis factor- alpha, Prostate cancer, Meta-analysis, Polymorphism

## Abstract

**Background:**

Tumor necrosis factor-alpha (TNF-α) is an important inflammatory cytokine that may play a role in controlling the progression of prostate cancer. Two common polymorphisms in the *TNF*-*α* gene, −308G/A and −238C/T, have been suggested to alter the risk for prostate cancer, but the results have been inconclusive so far. In order to obtain a better understanding of the effects of these two polymorphisms on prostate cancer risk, all available studies were considered in a meta-analysis.

**Methods:**

We conducted a comprehensive literature search in the Cochrane Library, PubMed, EMBASE, Chinese Biomedical Literature database (CBM), and the China National Knowledge Infrastructure (CNKI). The associations were evaluated by calculating the pooled odds ratio (OR) with 95% confidence interval (95% CI).

**Results:**

In this meta-analysis, we included 14 studies with 5,757 patients and 6,137 control subjects for the TNF-α-308G/A polymorphism and 1,967 patients and 2,004 control subjects for the TNF-α-238C/T polymorphism. A significantly increased prostate cancer risk was found to be associated with the TNF-α-308C/T polymorphism in studies with healthy volunteers (AA + AG vs. GG: OR = 1.531, 95% CI = 1.093–2.145; P = 0.013; AG vs. GG: OR = 1.477, 95% CI = 1.047–2.085; P = 0.026). No significant association was found between the TNF-α-238G/A polymorphism and prostate cancer risk in the overall or subgroup analyses. There was no risk of publication bias in this meta-analysis.

**Conclusions:**

Our results suggest that while the TNF-α-238G/A polymorphism may not be associated with prostate cancer the TNF-α-308C/T polymorphism may significantly contribute to prostate cancer susceptibility in healthy volunteers.

**Virtual slides:**

The virtual slide(s) for this article can be found here: http://www.diagnosticpathology.diagnomx.eu/vs/1629288120116301

## Introduction

Prostate cancer is the most common malignant tumour of the male reproductive system in the Western hemisphere. Approximately 238,590 new cases and 29,270 deaths of prostate cancer are reported annually [1]. Therefore, it is critically important to clarify the mechanism of its carcinogenesis, so that prostate cancer can be detected at an early stage. Polygenic and external environmental factors are known or suspected to be risk factors for prostate cancer [2-4]. Specifically, genetic factors may contribute as much as 42% to the risk of prostate cancer [5]. Recently, cytokine genetic polymorphisms were found to be related associated with increased inflammation, increased cytokine production, and possibly increased prostate cancer risk [6].

Tumour necrosis factor-alpha (TNF-α) is a mediator of the inflammatory process that is secreted by monocytes, macrophages, neutrophils, T cells, and NK cells after stimulation. TNF-α is a pro-inflammatory molecule that may play an important role in the development of the immune response [7,8] and affect the progression of prostate cancer [9]. The TNF-α gene is located in the major histocompatibility complex III region on chromosome 6p21.3. In recent years, several common single nucleotide polymorphisms (SNPs) have been identified in the TNF-α promoter regions, which can regulate the expression level of TNF-α, such as TNF-α-308G/A (rs1800629) and TNF-α-238C/T (rs361525) [10,11]. TNF-α 308G/A is a G to A transition at nucleotide position 308 in the promoter region of the gene, and TNF-α-238G/A is a G to A transition at nucleotide position 238 in the promoter region of the gene. Previous data have shown that polymorphisms of TNF-α at positions 308 and 238 (TNF2 and TNFA alleles, respectively) are associated with increased release of TNF-α [6,12,13]. Therefore, TNF-α polymorphism may be related to prostate cancer risk. Thus, it is biologically reasonable to hypothesize a potential relationship between TNF-α polymorphisms and prostate cancer risk.

A number of studies have reported the association between prostate cancer risk and TNF-α promoter polymorphisms –308G/A and/ or –238G/A polymorphisms, but the results are controversial [14-16]. A single study may not have sufficient power to completely demonstrate this complicated genetic relationship because of relatively small sample sizes, which have low statistical power. Larger studies could overcome these disadvantages. With respect to the TNF-α-308G/A polymorphism, Wang et al. [17] conducted a meta-analysis in 2011 and found that this polymorphism was not associated with susceptibility to prostate cancer. However, that meta-analysis only included six eligible studies and several new studies with more data have been published since 2011; thus, that meta-analysis may not be comprehensive and may cause some bias to the final result. To the best of our knowledge, no meta-analyses investigating the TNF-α-238G/A polymorphism have ever been published. Therefore, to generate a more valuable conclusion on the association between TNF-α polymorphisms and prostate cancer risk, we performed a meta-analysis of all eligible case–control studies investigating the association between the –308G/A and -238G/A polymorphisms of the TNF-α gene and prostate cancer risk.

## Methods

### Search strategy

We conducted a comprehensive literature search in the Cochrane Library, PubMed, EMBASE, Chinese Biomedical Literature database (CBM), and the China National Knowledge Infrastructure (CNKI) using the search terms “prostate cancer OR prostate carcinoma OR PCa”, “polymorphism or variant OR mutation OR genotype” and “Tumour Necrosis Factor-alpha OR TNF OR TNF-α” and various combinations of these terms. All the articles were updated on September 10, 2013. The search was performed without any limitations of language. Review articles, original articles, and other studies of interest were examined to identify additional eligible studies.

### Selection criteria

Studies included in the meta-analysis were required to meet the following criteria: (1) studies investigating the association between TNF-α gene polymorphisms and prostate cancer risk; (2) case–control or cohort studies; and (3) the papers must list the sample size, distribution of genotype, and allele frequency. If serial studies of the same population from the same group were reported, the most recent or largest population was chosen. When a study reported the results on different subpopulations, we treated it as separate studies in the meta-analysis. Studies were excluded from our meta-analysis if they met one of the following exclusion criteria: (1) the study was conducted on animals, (2) the design was based on family or sibling pairs, or (3) insufficient original data was available for data extraction, for instance, the number of genotypes could not be ascertained.

### Data extraction

T Two separate investigators (Ma and Zhao) reviewed and extracted data from the studies included independently to ensure the accuracy of the data. The following parameters were collected from eligible studies: first author's surname, year of publication, country of study population, ethnicity, genotyping methods, sample size, source of controls (hospital-based, population-based, or healthy volunteers), matching variables, prostate cancer diagnosis, QC when genotyping, and the number of genotype frequencies in cases and controls. The two investigators verified data accuracy by comparing collection forms between investigators. If different results were generated, they would carry out discussions until a consensus was reached.

### Quality score assessment

The quality of the selected studies was independently evaluated by two reviewers (Ma, and Zhao). The criteria for quality appraisal are listed in Table 1. The quality scoring system was originally proposed by Thakkinstian et al. [18]. Scores ranged from the lowest value 0 to the highest value 14, with higher scores indicating better quality. Disagreements were resolved by consensus.

**Table 1:** Scale for quality assessment

**Criteria**	**Score**
1. Representativeness of cases	
Selected from population or cancer registry	3
Selected from hospital	2
Selected from pathology archives, but without clearly defined sampling frame or with extensive inclusion/exclusion criteria	1
Not described	0
2. Source of controls	
Population- based	3
Blood donors or volunteers	2
Hospital-based (cancer -free patients)	1
Not described	0
3. Pecimens of cases determining genotypes	
White blood cells or normal tissues	1
Tumor tissues or exfoliated cells of tissue	0
4. Ascertainment of prostate cancer	
Histopathologic confirmation	2
Diagnosis of prostate cancer by patient medical record	1
Not described	0
5. Total sample size	
≧1000	3
≧400 but <1000	2
≧200 but <400	1
<200	0
6. Hardy-Weinbe rg equilibrium in controls	
Hardy-Wei nberg equilibrium	1
Hardy-Wei nberg disequilibrium	0
7. Quality control of genotyping methods	
Repetition of partial/total tested samples	1
Not described 0	0

### Statistical analysis

Crude odds ratios (ORs) and corresponding 95% confidence intervals (CIs) were employed to estimate the strength of association between the two polymorphisms and prostate cancer risk. The recessive genetic model, dominant genetic model and additive genetic models were used to calculate the pooled ORs and 95% CIs for both the polymorphisms. Heterogeneity among the studies included in the meta-analysis was evaluated by the chi-square-based Q test and quantified by the I^2^ metric. I^2^ values of 75%, 50%, and 25% were considered to reflect high, moderate, and low heterogeneity, respectively [19]. When no statistical heterogeneity was found (I^2^ < 50% or P > 0.10), the ORs and 95% CI would be estimated for each study in the fixed-effect model (Mantel-Haenszel method) [20]. Otherwise, the random-effect model (DerSimonian and Laird method) was applied [21]. Logistic meta-regression and subgroup analyses were performed to explore possible explanations for heterogeneity among studies. The following characteristics of participants were included as covariates in the meta-regression analysis: source of controls, ethnicity, genotyping methods, quality score, QC when genotyping, and prostate cancer diagnosis. Subgroup analyses were performed by ethnic group and source of controls. In addition, Galbraith plots analysis was performed for further exploration of heterogeneity.

A funnel plot was used to verify potential publication bias using the standard error of log (OR) for each publication plotted against its log (OR), and the asymmetry of the funnel plot was tested by Egger’s regression [22]. The *t* test was used to determine the significance of the asymmetry, and if the P value was <0.10, indicating the presence of publication bias, the non-parametric “trim and fill” method was used to adjust for it [23]. Sensitivity analysis was conducted to validate the credibility of outcomes in this meta-analysis. It was carried out by sequential omission of individual studies or by omitting studies without high quality. The Hardy-Weinberg equilibrium (HWE) in the controls was evaluated in our meta-analysis using the goodness-of-fit chi-square test, and p < 0.05 was considered representative of a departure from HWE. Statistical tests were performed using the program STATA 12.0 software (Stata-Corp LP, College Station, TX). All statistical tests were two-sided.

## Results

### Study characteristics

**Figure 1:**
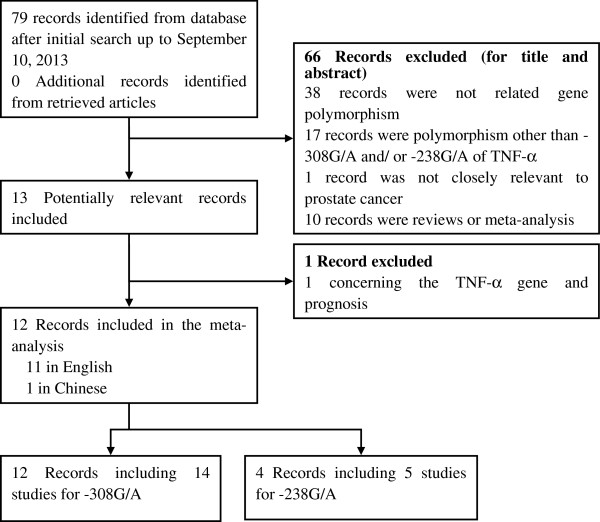
Flow diagram of included studies for this meta-analysis.

As shown in Figure 1, a total of 79 published records found on Cochrane Library, PubMed, EMBASE, CBM, and CNKI were identified as having met the search criteria. After the titles and abstracts were reviewed, 66 of these articles were excluded: 46 were not related to gene polymorphisms, 17 articles discussed polymorphisms other than –308G/A and/or –238G/A of TNF-α, 1 was not closely relevant to prostate cancer, and 2 were meta-analyses [17,24]. Manual search of references cited in the published studies did not reveal any more relevant articles. These 13 full-text articles were then subjected to further examination, and 1 article was further excluded as it discussed the TNF-α gene and prognosis. Thus, a total of 12 records with a case–control design met the inclusion criteria for the meta-analysis (Figure 1). Of these, two articles contained two different subpopulations and were treated as two independent studies. The corresponding characteristics are presented in Table 2. Twelve articles [14-16,25-33] with 14 studies (5,757 cases and 6,137 controls) discussed TNF-α-308G/A polymorphism, while 4 [15,16,30,31] articles containing 5 studies (1,967 cases and 2,004 controls) discussed TNF-α-238G/A polymorphism. The number of cases in these studies varied from 96 to 2,225, and the number of controls varied from 126 to 2,251. Of all the eligible studies for TNF-α-308G/A polymorphism, seven were performed in Caucasians populations; four, in Asians; two, in mixed populations (White and other); and one, in an African-American population. Similarly for TNF-α-238G/A polymorphism, three were performed in Caucasians and two in non-Caucasians. Genotyping was performed using polymerase chain reaction-restriction fragment length polymorphism (PCR-RFLP), amplification refractory mutation system-PCR (ARMS-PCR), TaqMan assay, and polymerase chain reaction (PCR) analyses on genomic DNA. For the TNF-α-308G/A polymorphism, the genotype distributions in the control groups of all but one study were in agreement with the HWE [33]. Similarly, for the TNF-α-238G/A polymorphism, the genotype distributions in the control group of one study [30] was not consistent with the HWE.

**Table 2:** **Characteristics of eligible studies in this meta**-**analysis**

**First author (year)**	**Country**	**Ethnicity**	**Genotyping methods**	**Source of controls**	**Sample size (case/control)**	**Matching variables**	**Prostate cancer diagnosis**	**QC when genotyping**	**PI**	**HWE (P value)**		**Quality scores**
										**−308G/A**	**−238G/A**	
Ribeiro (2012)	Portugal	Caucasia	TaqMan	HB	449/557	Age	HC	No	−308G/A	0.155	-	10
Berhane (2012)	India (N)	Asia	ARMS-PCR	HB	150/150	Age	HC	No	−308G/A	0.662	-	10
Wang (2009)	America (W)	mixed	TaqMan	PB	251/250	Age, sex,ethnicity	HC	No	−308G/A	0.529	-	12
Moore (2009)	Finland (W)	mixed	TaqMan	PB	949/857	Age, sex	HC	Yes	−308G/A	0.231	-	14
Kesarwani (2009)	India (E)	Asia	PCR-RFLP	PB	197/256	Age, sex, ethnicity	HC	Yes	−308G/A	0.115	-	13
Zabaleta 1 (2008)	America (W)	Caucasia	TaqMan	HB	950/785	Ethnicity	HC	Yes	−308G/A,-238G/A	0.505	0.295	10
Zabaleta 2 (2008)	America (W)	African-American	TaqMan	HB	131/256	Ethnicity	HC	Yes	−308G/A,-239G/A	0.959	0.006	8
Sáenz-López (2008)	Spain	Caucasia	TaqMa	HV	296/310	Ethnicity	HC	No	−308G/A	0.715	-	9
Danforth 1 (2008)	America (W)	Caucasia	TaqMan	PB	1155/1389	Age	HC	Yes	−308G/A	0.795	-	14
Danforth 2 (2008)	America (W)	Caucasia	TaqMan	PB	2225/2251	Age	HC	Yes	−308G/A,-239G/A	0.217	0.737	14
Ge (2007)	China	Asia	TaqMan	HB	490/490	Age	HC + PC	No	−308G/A	0.609	0.462	11
Wu (2004)	China	Asia	PCR	HV	96/126	Sex	HC	No	−308G/A,-239G/A	0.883	-	7
McCarron (2002)	UK	Caucasia	PCR	HB	239/220	Ethnicity	HC	No	−308G/A	0.023	-	7
OH (2000)	America	Caucasia	PCR	HB	146/244	Ethnicity	PC	Yes	−308G/A,-239G/A	0.554	0.091	9

PI, Polymorphism(s) investigated; QC, Quality control; NA, Not available; PB, Population–based; HB, Hospital–based; HV, healthy-volunteers; HC, Histologically confirmed; PC, Pathologically confirmed; HWE, Hardy–Weinberg equilibrium in control population.

### **Results of the meta**-**analysis**

**Figure 2:**
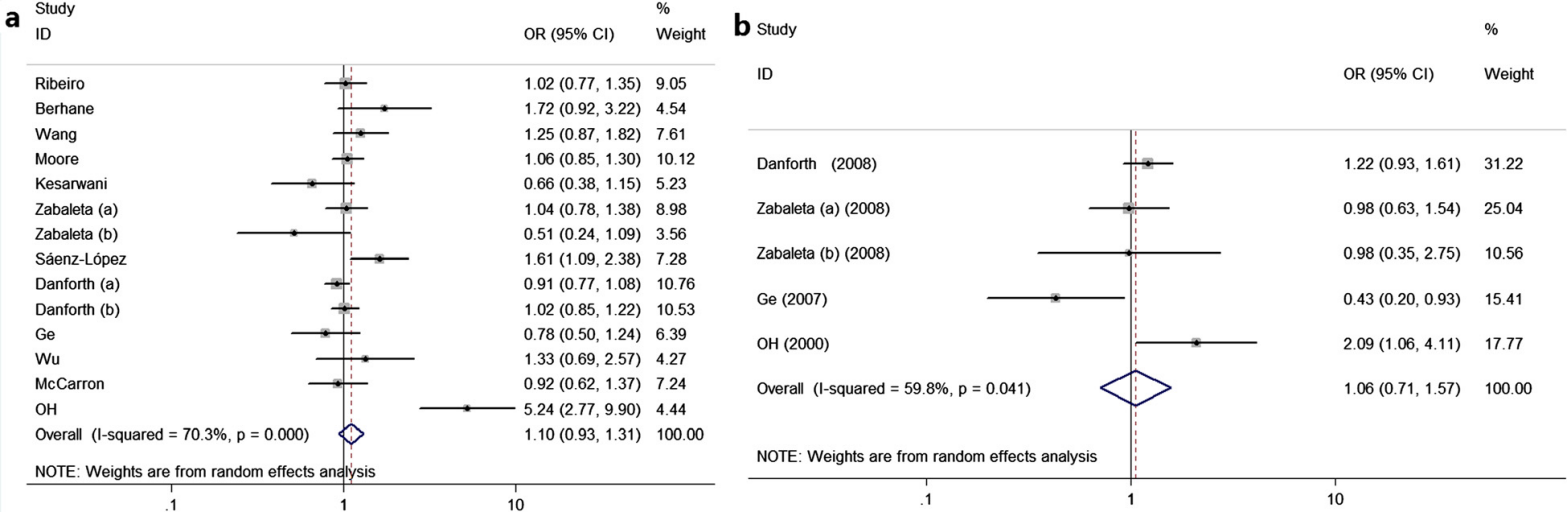
**Forest plots of TNF-α gene polymorphisms and prostate cancer risk. a** Forest plots of TNF-α-308G/A polymorphism and prostate cancer risk (AA + AG vs. GG), **b** Forest plots of TNF-α-238G/A polymorphism and prostate cancer risk (AA + AG vs. GG).

The data suggested no significant association between the TNF-α-308G/A polymorphism and prostate cancer risk in all genetic models (additive genetic models: AA vs. GG and AG vs. GG, recessive genetic model: AA vs. AG + GG, and dominant genetic model: AA + AG vs. GG; Table 3, Figure 2) in the overall populations. Additional subgroup analyses stratified by ethnicity revealed no association between TNF-α-308G/A polymorphism and prostate cancer risk in any of the genetic models. When stratified by source of controls, a significantly increased prostate cancer risk was found among healthy volunteer studies in the additive model AG vs. GG (OR = 1.477, 95% CI = 1.047–2.085, P = 0.026, I^2^ = 0.0%, and P_Q_ = 0.602 for heterogeneity) and dominant model AA + AG vs. GG (OR = 1.531, 95% CI = 1.093–2.145, P = 0.013, I^2^ = 0.0%, and P_Q_ = 0.628 for heterogeneity) but not in the recessive model AA vs. AG + GG (OR = 2.65, 95% CI = 0.679–10.341, P = 0.161, I^2^ = 0.0%, and P_Q_ = 0.997 for heterogeneity). Furthermore, excluding the studies by OH et al. [16] and Sáenz-López et al. [14], which were shown as outliers in our Galbraith plot analysis, did not influence the significance of the summary ORs for the TNF-α-308G/A polymorphism in different comparison models in the overall population and subgroup analyses.

**Table 3:** **Meta**-**analysis of the TNF**-**α gene polymorphisms on prostate cancer risk**

**Comparison**	**Population**	**No. of studies**	**Test of association**				**Test of heterogeneity**		
			**OR**	**95% CI**	**P value**	**model**	**X2**	**I2(%)**	**P value**
TNF-α-308G/A									
AA vs. GG	Overall	14	0.936	0.733-1.195	0.595	F	16.20	19.8	0.238
	Caucasia	8	0.762	0.569-1.021	0.068	F	5.68	0.0	0.461
	Asia	3	1.558	0.620-3.419	0.346	F	4.29	30.1	0.231
	Mix	2	1.616	0.917-2.848	0.097	F	0.11	0.0	0.736
	African-American	1	1.119	0.181-6.903	0.904	-	-	-	-
	PB	5	0.886	0.660-1.190	0.422	F	6.72	40.5	0.152
	HB	7	0.924	0.557-1.481	0.743	F	6.76	11.2	0.344
	HV	2	2.861	0.732-11.181	0.131	F	0.00	0.0	0.979
AG vs. GG	Overall	14	1.105	0.932-1.309	0.251	R	41.38	68.6	<0.001
	Caucasia	7	1.234	0.965-1.600	0.092	R	31.63	81.0	<0.001
	Asia	4	0.928	0.699-1.234	0.608	F	4.12	27.2	0.249
	Mix	2	1.065	0.882-1.288	0.512	F	0.70	0.0	0.404
	African-American	1	0.458	0.204-1.027	0.058	-	-	-	-
	PB	5	0.997	0.899-1.106	0.958	F	3.39	0.0	0.495
	HB	7	1.174	0.709-1.746	0.428	R	32.78	81.7	<0.001
	HV	2	1.477	1.047-2.085	0.026	F	0.27	0.0	0.602
AA + AG vs. GG	Overall	14	1.103	0.932-1.306	0.254	R	43.71	70.3	<0.001
	Caucasia	8	1.206	0.941-1.545	0.138	R	32.21	81.4	<0.001
	Asia	3	1.008	0.657-1.547	0.97	R	6.83	56.1	0.078
	Mix	2	1.101	0.916-1.323	0.304	F	0.63	0.0	0.429
	African-American	1	0.513	0.242-1.088	0.082	-	-	-	-
	PB	5	0.987	0.892-1.091	0.799	F	4.96	19.4	0.291
	HB	7	1.174	0.803-1.719	0.408	R	32.22	81.4	<0.001
	HV	2	1.531	1.093-2.145	0.013	F	0.24	0.0	0.628
AA vs. GG + AG	Overall	14	0.92	0.721-1.172	0.502	F	16.2	19.7	0.239
	Caucasia	7	0.748	0.559-1.001	0.051	F	5.93	0.0	0.431
	Asia	4	1.559	0.618-3.935	0.347	F	3.98	24.7	0.263
	Mix	2	1.569	0.893-2.757	0.117	F	0.19	0.0	0.660
	African-American	1	1.303	0.212-7.991	0.775	-	-	-	-
	PB	5	0.883	0.659-1.184	0.407	F	6.26	36.1	0.181
	HB	7	0.886	0.555-1.413	0.611	F	7.49	19.9	0.278
	HV	2	2.65	0.679-10.341	0.161	F	0.00	0.0	0.997
TNF-α-238G/A									
AA vs. GG	Overall	4	1.09	0.403-2.943	0.866	F	4.61	34.9	0.203
	Caucasia	3	1.29	0.436-3.816	0.645	F	4.35	54.1	0.113
	African-American	1	0.391	0.018-8.288	0.547	-	-	-	-
	HB	3	1.579	0.492-5.071	0.442	F	3.750	46.7	0.153
	PB	1	0.344	0.036-3.310	0.355	-	-	-	-
AG vs. GG	Overall	5	1.07	0.680-1.683	0.77	R	12.49	68.0	0.014
	Caucasia	3	1.284	0.841-2.025	0.282	R	5.99	66.6	0.050
	Asia	1	0.431	0.200-0.931	0.032	-	-	-	-
	African-American	1	1.179	0.408-3.405	0.706	-	-	-	-
	HB	4	1.008	0.506-2.010	0.981	R	11.3	73.4	0.01
	PB	1	1.248	0.944-1.649	0.12	-	-	-	-
AA + AG vs. GG	Overall	5	1.06	0.713-1.575	0.774	R	9.95	59.8	0.041
	Caucasia	3	1.226	0.982-1.531	0.072	F	3.33	39.9	0.189
	Asia	1	0.431	0.200-0.931	0.032	-	-	-	-
	African-American	1	0.983	0.351-2.752	0.974	-	-	-	-
	HB	4	0.984	0.535-1.808	0.958	R	9.170	67.3	0.027
	PB	1	1.221	0.926-1.610	0.156	-	-	-	-
AA vs. GG + AG	Overall	4	1.036	0.388-2.767	0.943	F	4.9	38.2	0.182
	Caucasia	3	0.892	0.084-9.494	0.925	R	4.63	56.8	0.099
	African-American	1	0.386	0.018-8.161	0.541	-	-	-	-
	HB	3	1.468	0.467-4.615	0.511	F	4.050	50.6	0.132
	PB	1	0.336	0.035-3.238	0.346	-	-	-	-

Data from five case–control studies comprising 1,967 prostate cancer cases and 2,004 controls were pooled for the analysis of the TNF-α-238G/A polymorphism. No association was detected between TNF-α-238G/A polymorphism and prostate cancer risk in all genetic models in the overall population. Similarly, in the subgroup analysis by source of controls and ethnicity, no significant association was found (Table 3).

### Heterogeneity analysis

For the TNF-a-308G/A polymorphism, substantial heterogeneities between studies were observed in the additive and dominant models (p < 0.001 in both models) in the overall populations. We further employed meta-regression and subgroup analyses to explore the source of heterogeneity. However, meta-regression analysis of data showed that ethnicity, genotyping methods, source of controls, QC when genotyping, prostatic cancer diagnosis, and quality scores did not affect modifiers. Subsequently, we stratified the studies by ethnicity and source of controls, and observed the lack of homogeneity among Caucasians and hospital-based studies in the two genetic models (Table 3). Ethnicity and different source of controls were significant factors for heterogeneity.

**Figure 3:**
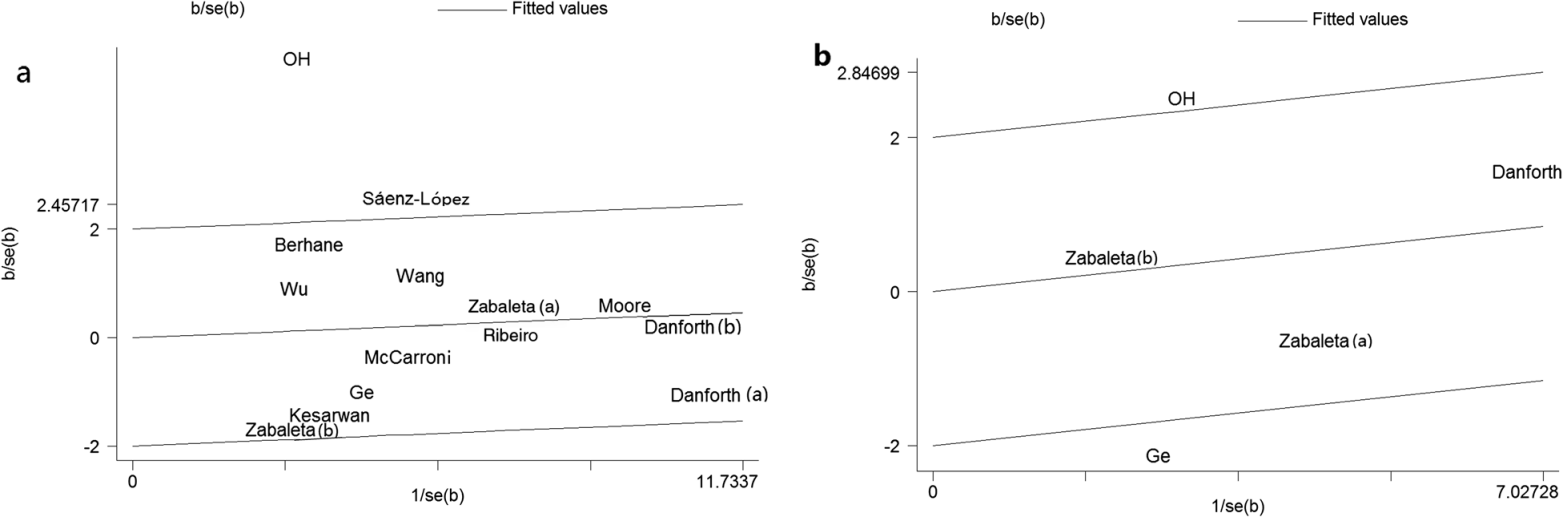
**Galbraith plots of TNF-α-308G/A polymorphism and prostate cancer risk in different contrast models. a** The studies of OH et al., Sáenz-López et al. were outliers in dominant models AA + AG vs. GG. **b** OH et al. was outlier in additive model AG vs. GG.

 We also performed Galbraith plots analysis to identify the outliers that might contribute to the heterogeneity. The study of Oh et al. [16] was an outlier in the additive model AG vs. GG, while the studies of Oh et al. [16] and Sáenz-López et al. [14] were both outliers in the dominant model AA + AG vs. GG for TNF-α-308G/A polymorphism (Figure 3). When these two studies were excluded from the analysis, the I^2^ values decreased obviously and the P_Q_ values were greater than 0.10 in the overall populations (additive model: P_Q_ = 0.267, I^2^ = 17.5%; dominant model: P_Q_ = 0.295, I^2^ = 15.2%), Caucasians (additive model: P_Q_ = 0.379, I^2^ = 5.9%; dominant model: P_Q_ = 0.883, I^2^ = 0.0%), and hospital-based studies (additive model: P_Q_ = 0.294, I^2^ = 18.4%; dominant model: P_Q_ = 0.240, I^2^ = 25.9%).

For the TNF-α-238G/A polymorphism, substantial heterogeneities were observed between studies in the additive model AG vs. GG (p = 0.014) and dominant model AA + AG vs. GG (p = 0.041) in the overall populations. We further employed subgroup analyses stratified by ethnicity and source of controls. However, heterogeneities were still observed among hospital-based studies in these two genetic models (Table 3). Therefore, ethnicity was considered to contribute to substantial heterogeneity.

### Sensitivity analysis

A leave-one-out sensitivity analysis was performed to reflect the influence of the individual dataset to the pooled ORs. The results suggested that no single investigation significantly affected the pooled ORs (data not shown). When we excluded HWE-violating studies, the corresponding pooled ORs were not materially altered, indicating that the data in this meta-analysis are relatively stable and credible.

### Publication bias

**Figure 4:**
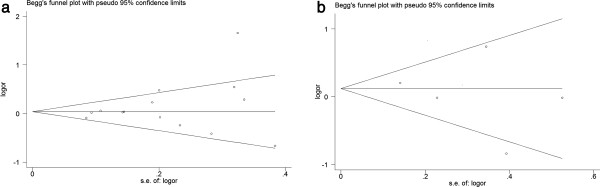
**Funnel plot for the detection of the publication bias in this meta-analysis. a** Funnel plot for contrast AA + AG vs. GG of TNF-α-308G/A polymorphism in overall analysis; **b** Funnel plot for contrast AA + AG vs. GG of TNF-α-238G/A polymorphism in overall analysis.

Begg’s funnel plot and Egger's linear regression tests were created to estimate the publication bias risk in this meta-analysis. As shown in Figure 4, the shapes of the funnel plots did not show obvious asymmetry. In addition, the results of Egger’s test also revealed the absence of publication bias in the TNF-α-308G/A (P = 0.224 for AA vs. GG; P = 0.350 for AG vs. GG; P = 0.313 for recessive model AA vs. GG + AG; and P = 0.275 for dominant model AA + AG vs. GG) and TNF-α-238G/A (P = 0.742 for AA vs. GG; P = 0.758 for AG vs. GG; P = 0.763 for recessive model AA vs. GG + AG; and P = 0.629 for dominant model AA + AG vs. GG) polymorphisms.

## Discussion

TNF-α is a member of the TNF/TNFR cytokine superfamily, and is an intercellular communicating molecule involved in building transient or long-lasting multicellular structures [34]. It is also known to play critical and non-redundant roles in the innate and adaptive immune responses [35], including the response to tumours [36]. TNF-α has been directly and indirectly linked to neoplasia and is involved not only in maintenance and homeostasis of the immune system, inflammation, and host defence, but also in pathological processes such as chronic inflammation, autoimmunity, and malignant disease [35,37]. TNF-α expression is mostly regulated at the transcriptional level [38]. Promoter polymorphisms in the TNF-α gene related to the pro- and anti-inflammatory response could directly influence production of TNF-α, thus causing inter-individual differences in immune responsiveness, which may influence the susceptibility of prostate cancer [39].

Two variations in the promoter region of the TNF-α gene, namely, TNF-α-308G/A and TNF-α-238G/A, have been commonly studied. The G to A substitution at position −308 in the TNF-a promoter increases TNF-α transcription activity and the serum TNF-α level [6]. Indeed, the TNF-308A allele has been associated with malignant tumours such as gastric cancer, breast carcinoma, and hepatocellular cancer [40-42]. The functional significance of the rare TNF-238A allele is not yet clear, but Kaluza et al. reported that this allele caused a significant decreased in the transcription of the TNF-α gene in human T and B cells [43]. The allele has also been associated with certain autoimmune and infectious diseases [44,45]. Several studies have observed the association between prostate cancer risk and TNF-α promoter polymorphisms, and TNF-α-308G/A and/or TNF-α-238G/A polymorphisms, but the results are controversial. These inconsistent results are possibly because of the small effect of the polymorphism on prostate cancer risk or the relatively low statistical power of the published studies. Meta-analysis could overcome these disadvantages because (1) it can investigate data for a large number of individuals; (2) it can estimate the effect of a genetic factor on disease risk; and (3) if a significant association is found, it can estimate whether the association is common among different backgrounds (such as population or age groups) [46-49]. Therefore, we performed a meta-analysis to comprehensively evaluate the association between TNF-α-308G/A and/or TNF-α-238G/A polymorphisms and prostate cancer risk.

Our meta-analysis summarized for the first time all the available data on the association between TNF-α-238G/A polymorphism and prostate cancer risk, including a total of five studies, involving 1,967 prostate cancer cases and 2,004 controls. Our results demonstrated that the TNF-α-238G/A polymorphism was not significantly associated with prostate cancer risk not only in the overall population but also in the subgroup analyses stratified by ethnicity and source of controls.

With respect to TNF-α-308G/A polymorphism, 14 studies including 5,757 prostate cancer cases and 6,137 controls were found in our meta-analysis. The data suggested no significant association between TNF-α-308G/A polymorphism and prostate cancer risk in all genetic models in the overall populations, which is consistent with the previous findings made by Wang et al. [24] and Wang et al. [17]. However, in the subgroup analysis according to source of controls, significantly increased prostate cancer risk was found in the healthy volunteer studies but not in hospital-based and population-based studies. However, the result may be underpowered because the sample size the healthy volunteer studies in this analysis is relatively small, and controls in these studies may not always be truly representative of the general population. Therefore, a methodologically preferable design such as a representative population-based study is needed to avoid selection bias and to increase the statistical power.

For the TNF-α-308G/A polymorphism, substantial heterogeneities between studies were observed in the additive model AG vs. GG (p < 0.001) and dominant model AA + AG vs. GG (p < 0.001) in the overall populations. To explore the source of heterogeneity, we employed meta-regression and subgroup analyses. Meta-regression analysis revealed no definite source of heterogeneity. Subgroup analyses by ethnicity showed that heterogeneity existed in Caucasian subjects and hospital-based studies in the additive and dominant models. To further investigate the heterogeneity, Galbraith plot analysis was used to identify the outliers that might contribute to the heterogeneity, and two outliers were found. When they were excluded from the analysis, the I^2^ values decreased obviously and P_Q_ values were greater than 0.10 in the overall populations and in Caucasians. In addition, excluding the two studies did not significantly affect the results in the different comparison models in the overall population and subgroup analyses. The results indicated that the two studies might be the major source of the heterogeneity for the 308G/A polymorphism.

Substantial heterogeneities between studies were observed in the additive and dominant models for the TNF-α-238G/A polymorphism in the Caucasian population and hospital-based studies. As only five studies on the association between TNF-α-238G/A polymorphism and prostate cancer risk were included in our meta-analysis, meta-regression analysis and Galbraith plots analysis were not performed; therefore, these results require further investigation.

This meta-analysis has some limitations that must be considered. First, the overall outcomes were based on individual unadjusted ORs, while a more precise estimation should be adjusted by confounding factors such as smoking status, age, and environmental factors. Second, the sample sizes in this analysis were not adequate, especially the African-American populations; therefore, more subjects of different ethnicities would be required to accurately clarify whether ethnicity has a biological influence on cancer susceptibility. Third, the controls were not consistently screened across the studies analysed. Therefore, the control groups may have different risks of developing prostate cancer. Fourth, as only certain published studies were included in our meta-analysis, publication bias is very likely to occur although it was not shown in the statistical test.

## Conclusions

In conclusion, the present meta-analysis suggests that the TNF-α-238/A polymorphism is unlikely to be a risk factor for prostate cancer, while the TNF-α-308 G/A polymorphism may make a significant contribution to the risk of prostate cancer in healthy volunteers. However, to clarify the role of TNF-α-308G/A and TNF-α-238G/A polymorphism in prostate carcinogenesis, more studies with large samples are needed in the future.

## Abbreviations

TNF-α: Tumor necrosis factor-alpha; SNPs: Single nucleotide polymorphisms; HWE: Hardy–Weinberg equilibrium; OR: Odds ratio; CI: Confidence interval; PCR-RFLP PCR: Based restriction fragment length polymorphism; QC: Quality control.

## Competing interests

The authors declare that they have no competing interests.

## Authors’ contributions

All authors have read and approved the final files for this manuscript.
